# Unicompartmental vs. segmental bicompartmental vs. total knee replacement: comparison of clinical outcomes

**DOI:** 10.1186/s43019-020-00065-0

**Published:** 2020-08-31

**Authors:** Oday Al-Dadah, Georgina Hawes, Philip J. Chapman-Sheath, John William Tice, David S. Barrett

**Affiliations:** 1 Translational and Clinical Research Institute, Newcastle University, Framlington Place, Newcastle-upon-Tyne, NE2 4HH UK; 2 Department of Trauma and Orthopaedic Surgery, South Tyneside Hospital, Harton Lane, South Tyneside, NE34 0PL UK; 3 Department of Trauma and Orthopaedic Surgery, University Hospital Southampton, Tremona Road, Southampton, Hampshire SO16 6YD UK; 4 School of Engineering Sciences, University of Southampton, Highfield, Southampton, SO17 1BJ UK

**Keywords:** Bicompartmental, Unicompartmental, Total knee replacement, Arthroplasty, Clinical outcome scores

## Abstract

**Purpose:**

Combined medial tibiofemoral and symptomatic patellofemoral osteoarthritis is not amenable to unicompartmental knee replacement (UKR). Total knee replacement (TKR) is an invasive option in younger adults with high functional demands. The aim of this study was to compare the clinical outcome of patients who have undergone UKR, bicompartmental knee replacement (BKR) and TKR up to 2 years post-operatively.

**Materials and methods:**

This prospective study comprised 133 subjects including 30 patients in the medial UKR group, 53 patients in the BKR group (combined medial UKR with patellofemoral joint replacement) and 50 patients in the TKR group. All subjects were evaluated using the Oxford Knee Score (OKS) and the Western Ontario and MacMaster Universities Osteoarthritis Index (WOMAC). Patients in each group were assessed using both scoring systems pre-operatively and 6 months, 1 year and 2 years post-operatively.

**Results:**

Significant improvement of OKS was found at 6 months compared to baseline for UKR (22.7 to 38.1, *p* = 0.046), BKR (22.6 to 36.8, *p* < 0.001) and TKR (16.6 to 34.5, *p* < 0.001). Significant improvement was also found for the WOMAC sub-scores for all three groups during this time period. After 6 months, there was no further statistically significant improvement in either outcome score in any of the groups up to the 2-year follow-up results. There was no significant difference in either outcome score post-operatively between the three groups.

**Conclusion:**

The magnitude of clinical improvement following knee replacement is greatest at 6 months; thereafter, only modest improvements continue to occur. This study also found no significant differences of outcomes at 2 years after surgery among UKR, BKR and TKR. BKR is a good alternative option for combined symptomatic medial and patellofemoral arthritis of the knee.

## Introduction

The management of young adults with symptomatic osteoarthritis of the knee that is refractory to conservative measures is challenging. Total knee replacement (TKR) can successfully treat arthritis albeit at the expense of bone stock and intra-articular ligaments. This can represent theoretical disadvantages, particularly for young patients with high functional demands and high risk for potential revision surgery within their lifetime. Unicompartmental knee replacement (UKR) has been shown to be an effective surgical option in patients with localised disease confined to one compartment that can obviate the need for TKR, with studies reporting up to 91% survivorship at 20 years [1]. However, medial tibiofemoral compartment arthritis in the presence of generalised patellofemoral joint (PFJ) arthritis (particularly involving the lateral patellar facet) yields poorer clinical results when treated by UKR alone [2, 3]. Furthermore, studies have shown that the main indication for revision of UKR is progression of PFJ arthritis [4, 5]. Arthritis progression following UKR surgery can be minimised by a careful patient selection criteria and avoiding deformity overcorrection in the coronal plane [6]. PFJ replacement also has a role in the management of isolated PFJ arthritis. Although earlier PFJ prostheses were noted to have poor outcomes [7] more recent studies have shown substantially better results [8–10]. One of the commonest indications for PFJ revision surgery is osteoarthritic progression of the tibiofemoral compartments [7, 11]. The improvement of PFJ replacement surgery is attributable to improved implant designs, better patient selection and improved surgical technique [12].

 Bicompartmental osteoarthritis disease pattern is a relatively common finding in the knee [13]. When the disease process involves more than one compartment then the situation becomes less favourable for UKR or PFJ replacement alone. These patients may be better served by a segmental bicompartmental knee replacement (BKR). Bicompartmental arthroplasty (a combination of UKR plus PFJ replacement) is less invasive than TKR and is a bone-stock and cruciate-ligament-sparing procedure. This may give a more ‘physiological’ knee that more closely approximates normal kinematics [13]. BKR can be a successful approach to prevent or postpone the need for TKR. However, this intervention is technically demanding and requires training and experience in both UKR and PFJ replacement surgery. Revision of TKR is a particularly invasive procedure that requires the use of large revision implants. Revision of BKR is comparatively easier as conversion to a standard primary TKR is an available fallback option if needed [14]. This might make BKR an attractive alternative, especially for the young active patient with a high likelihood of future revision.

The literature to date is sparse regarding clinical outcome data of BKR. The limited available literature has been complicated by studies coalescing the results of combined medial and lateral UKRs, medial UKR plus PFJ replacement, lateral UKR plus PFJ replacement and monolithic (femoral monobloc) prostheses all being analysed together. Previous reports [11, 13, 15, 16] of this concept of partial replacement surgery have suggested a significant difference in the survival between these different surgical procedures. We propose a separate modular bicompartmental classification of bi-unicompartmental (combined medial and lateral UKRs), medial segmental (medial UKR plus PFJ replacement) and lateral segmental (lateral UKR plus PFJ replacement) to accurately distinguish between these different surgical techniques and allow greater clarification in the literature when comparing results of studies.

The aim of this study was to compare the clinical outcome of patients who have undergone medial UKR, medial segmental BKR and TKR. We hypothesised that the outcome of BKR would be similar to that of UKR and TKR.

## Material and methods

### Patients

The study was a prospective, comparative cohort study assessing the clinical outcomes of patients who underwent UKR, BKR and TKR in our department. This study was exempt from Institutional Review Board (IRB) approval as it was a pragmatic study evaluating the existing clinical practice of each of the surgeons included in the study. There was a total of 133 subjects included in the study. This comprised 30 patients in the UKR group, 53 patients in the BKR group and 50 patients in the TKR group. Table 1 shows the demographics for the subjects in each group. Written informed consent was obtained from all the participants. All patients who underwent surgery had symptomatic osteoarthritis that was refractory to prior conservative treatment. All cases involved unilateral surgery. All patients were mobilised full weight-bearing with full range of movement (ROM) as tolerated and underwent a standardised post-operative physiotherapy rehabilitation programme. All the cases included in this study had their surgery performed under the care of one of three fellowship trained consultant orthopaedic surgeons with a specialist interest in knee surgery who worked in a tertiary referral university hospital. There were no revision surgical procedures in any of the three groups within the study time period from 2009 to 2012.

**Table 1:** Demographics of subjects

	UKR^a^	BKR^b^	TKR^c^
	(*n* = 30)	(*n* = 53)	(*n* = 50)
Mean age (years) (SD)	60 (11)	55 (9)	59 (7)
Male: female	13:17	22:31	16:34
Index knee (right: left)	17:13	31:22	26:24

^a^*UKR* unicompartmental knee replacement group

^b^*BKR* bicompartmental knee replacement group

^c^*TKR* total knee replacement group

The patients in the UKR group had isolated grade-IV medial tibiofemoral arthritis with intact cruciate ligaments and no associated articular cartilage lesions within the remaining two compartments of the knee greater than grade II (superficial fibrillation) of the modified Outerbridge classification [17–19]. The medial UKR prostheses (selected by individual surgeon’s preference) included the Sigma High Performance (HP) UKR (DePuy, Warsaw, IN, USA) and the High Flex UKR (Zimmer, Warsaw, IN, USA). Both prostheses are cemented with a metal-backed, fixed-bearing tibial component. All UKR procedures were performed through a minimally invasive, quadriceps-sparing surgical approach.

**Fig. 1:**
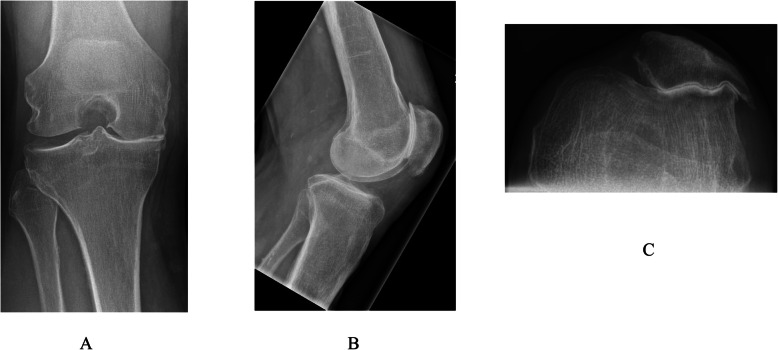
Knee radiographs of ideal candidate for medial segmental bicompartmental knee replacement. **a** Rosenberg **b** Lateral **c** Skyline

**Fig. 2:**
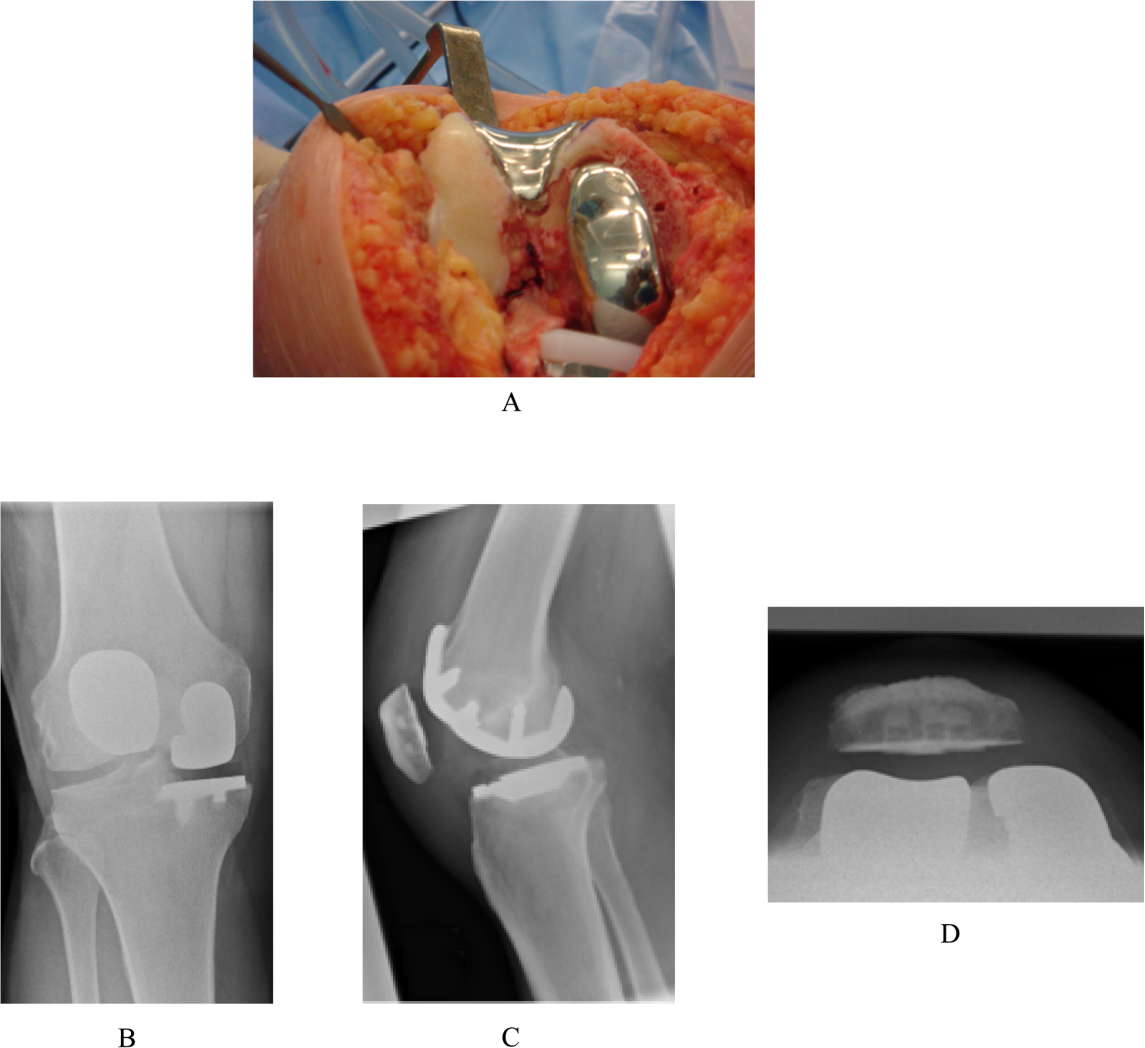
Sigma High Performance unicompartmental knee replacement (UKR) and patello-femoral joint (PFJ) medial segmental bicompartmental knee replacement prostheses (DePuy, Warsaw, IN, USA). **a** Clinical photograph. Knee radiographs **b** Antero-posterior weight-bearing **c** Lateral **d** Skyline

**Fig. 3:**
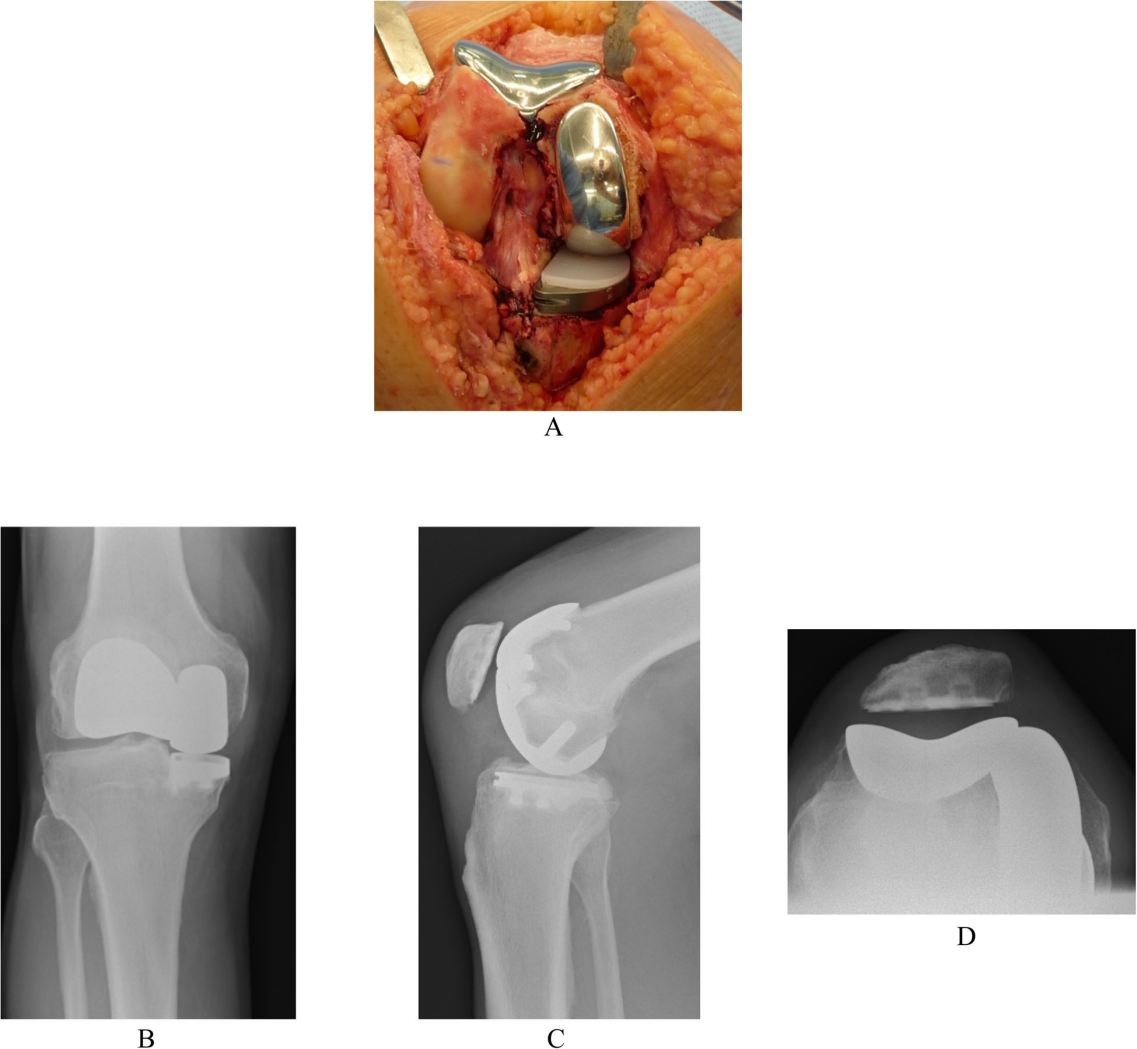
High Flex unicompartmental knee replacement (UKR) and Gender Solutions patello-femoral joint (PFJ) medial segmental bicompartmental knee replacement prostheses (Zimmer, Warsaw, IN, USA). **a** Clinical photograph. Knee radiographs **b** Antero-posterior weight-bearing **c** Lateral **d** Skyline

Similarly, the BKR group had intact cruciate ligaments but with grade-IV arthritis affecting both the medial tibiofemoral and patellofemoral compartments but with well-preserved articular cartilage (no greater than grade II) of the lateral tibiofemoral compartment (Fig. 1). They underwent simultaneous segmental medial UKR and PFJ replacement at the same primary surgical procedure. The indication for PFJ replacement included grade-IV arthritis visible on skyline view x-ray with associated anterior knee pain, palpable crepitus and a positive patellar grind test. The patients who received the Sigma HP UKR prosthesis also received the Sigma HP PFJ replacement (DePuy, Warsaw, IN, USA) (Fig. 2). The patients who received the High Flex UKR prosthesis also received the Gender Solutions PFJ replacement (Zimmer, Warsaw, IN, USA) (Fig. 3). Both PFJ prostheses consisted of cemented trochlea and polyethylene patella resurfacing components. All BKR procedures were performed through a medial parapatellar surgical approach using a notably smaller skin incision than that for TKR.

The TKR group had widespread grade-IV degenerative changes affecting all three compartments of the knee. The TKR prostheses (selected by individual surgeon’s preference) included the PFC Sigma HP TKR (DePuy, Warsaw, IN, USA) and the NexGen TKR (Zimmer, Warsaw, IN, USA). Both were cemented, fixed-bearing, posterior-stabilised prostheses. All patients in the TKR group also underwent cemented polyethylene patella resurfacing to minimise possible confounding factors as compared to the BKR group in particular. All TKR procedures were performed through a standard medial parapatellar surgical approach.

### Patient-reported outcome measures (PROMs)

Patients in all three groups were assessed using two validated clinical outcome knee scoring systems pre-operatively and 6 months, 1 year and 2 years post-operatively. These included the Oxford Knee Score (OKS, using the new (0–48) scoring system) [20, 21] and the Western Ontario and MacMaster Universities Osteoarthritis Index (WOMAC) [22].

### Statistical analysis

Plotted histograms and the Kolmogorov-Smirnov test were used to confirm that a Normal distribution was an appropriate assumption for all the variables in the study. One-way analysis of variance (ANOVA) and Tukey’s post-hoc pairwise comparison was used for the purpose of between-group statistical analyses. The paired Student’s *t* test was used for the longitudinal within-group analyses. The level of statistical significance was set at *p* < 0.05. Statistical analysis was performed using SPSS for Windows version 20.0 (IBM Corp., Armonk, New York).

## Results

### Oxford Knee Score

**Fig. 4:**
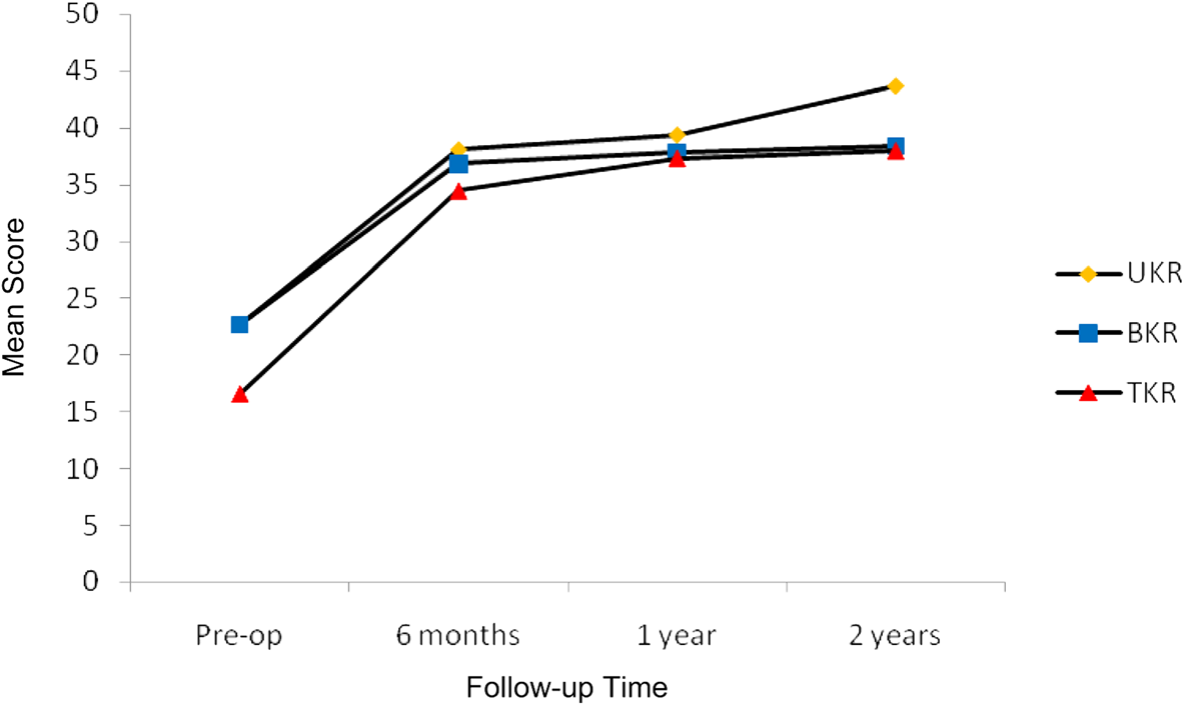
Mean Oxford Knee Scores. Significant improvement of 6-month post-operative results as compared to pre-operative findings in all three groups. Thereafter, no further statistically significant longitudinal improvement within any of the groups at any time point

The mean scores of the OKS for each of the three groups are displayed in Fig. 4. The results of the longitudinal within-group statistical analysis in Table 2 revealed a significant improvement of the mean 6-month post-operative results as compared to the baseline pre-operative findings for all three groups. After 6 months there was no further statistically significant longitudinal improvement within any of the groups up to the 2-year follow-up results. It was not possible to perform the analysis for the UKR group at the 1-year vs. 2-year time point as it was underpowered due to missing data points and loss to follow-up. The between-group statistical analysis shown in Table 3 revealed that pre-operatively there was no significant difference of the OKS between the UKR group and the BKR group. However, there was a significant difference when the TKR group was compared to the UKR group and also the BKR group pre-operatively. Post-operatively there was no significant difference of the mean OKS between any of the three groups at any time point.

**Table 2:** Within-group statistical analysis of Oxford Knee Scores

	Pre-operative vs. 6 months		6 months vs. 1 year		1 year vs. 2 years	
	*p* value	95%CI	*p* value	95%CI	*p* value	95%CI
UKR^a^	0.046^*^	0.4 to 30.8	0.336	− 5.7 to 10.3	N/A	
BKR^b^	< 0.001^*^	11.3 to 19.9	0.071	− 0.3 to 4.8	0.635	− 17.5 to 13.5
TKR^c^	< 0.001^*^	13.6 to 21.9	0.535	− 2.2 to 4.1	0.078	− 0.3 to 3.5

Paired Student’s *t* test

*95%CI* confidence interval of mean difference

*N/A* not analysed as underpowered

^*^Statistically significant at < 0.05 level

^a^*UKR* unicompartmental knee replacement group (6 months: *n* = 21; 1 year: *n* = 15; 2 years: *n* = 4)

^b^*BKR* bicompartmental knee replacement group (6 months: *n* = 39; 1 year: *n* = 26; 2 years: *n* = 15)

^c^*TKR* total knee replacement group (6 months: *n* = 41; 1 year: *n* = 29; 2 years: *n* = 11)

**Table 3:** Between-group statistical analysis of Oxford Knee Scores

	UKR^a^ vs. BKR^b^		UKR^a^ vs. TKR^c^		BKR^b^ vs. TKR^c^	
	*p* value	95%CI	*p* value	95%CI	*p* value	95%CI
Pre-operative	1.000	− 5.8 to 5.9	0.012*	1.1 to 11.1	0.013*	1.1 to 11.0
6 months	0.917	− 6.7 to 9.3	0.461	− 3.7 to 10.9	0.641	− 8.4 to 3.8
1 year	0.899	− 7.1 to 10.3	0.824	− 6.3 to 10.3	0.986	− 6.3 to 7.2
2 years	0.497	− 6.4 to 17.0	0.478	− 6.5 to 17.9	0.993	− 8.7 to 8.0

One-way analysis of variance (ANOVA) and Tukey’s post-hoc pairwise comparison

*95%CI* confidence interval of mean difference

^*^Statistically significant at < 0.05 level

^a^*UKR* unicompartmental knee replacement group (6 months: *n* = 21; 1 year: *n* = 15; 2 years: *n* = 4)

^b^*BKR* bicompartmental knee replacement group (6 months: *n* = 39; 1 year: *n* = 26; 2 years: *n* = 15)

^c^*TKR* total knee replacement group (6 months: *n* = 41; 1 year: *n* = 29; 2 years: *n* = 11)

### Western Ontario and MacMaster Universities Osteoarthritis Index (WOMAC)

**Fig. 5:**
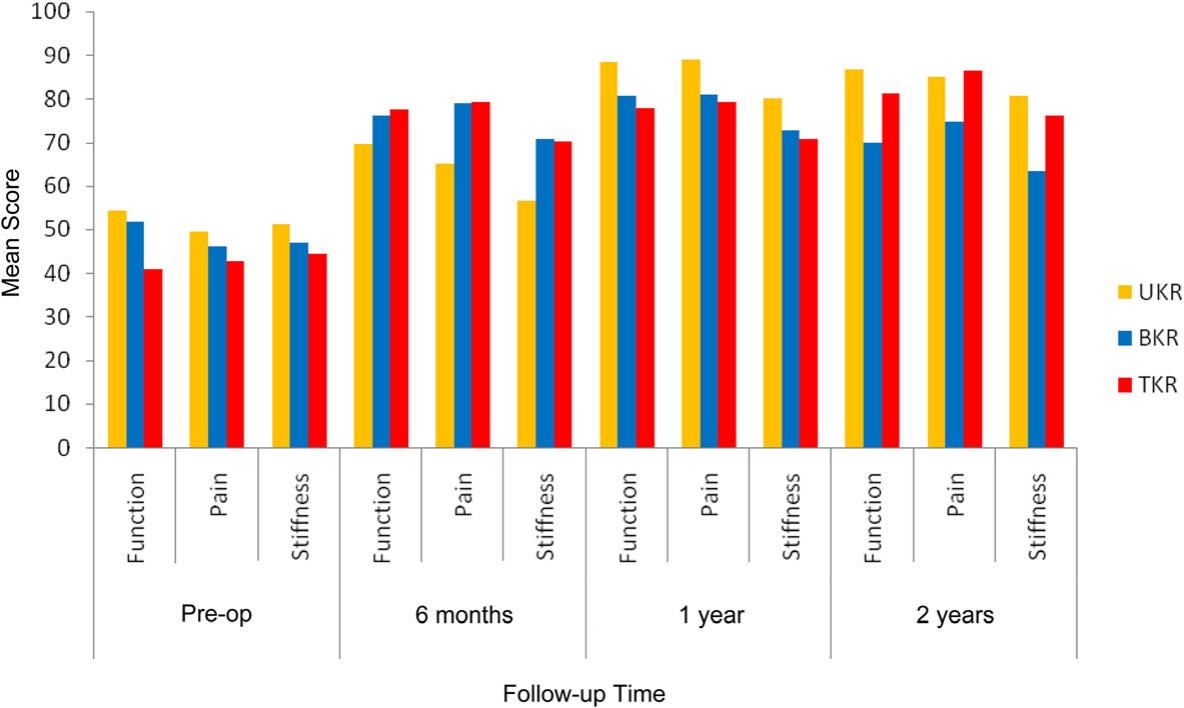
Mean Western Ontario and MacMaster Universities Osteoarthritis Index (WOMAC) sub-scores. Overall a significant improvement of 6-month post-operative results as compared to pre-operative findings for the bicompartmental knee replacement (BKR) and total knee replacement (TKR) groups but not the unicompartmental knee replacement (UKR) group. Thereafter, no further statistically significant longitudinal improvement generally within any of the groups at any time point

The mean WOMAC sub-scores for each of the three groups is displayed in Fig. 5. The results of the longitudinal within-group statistical analysis in Table 4 revealed a significant improvement of the mean 6-month post-operative results as compared to the baseline pre-operative findings for the BKR and TKR groups. The UKR group revealed a borderline significant improvement of the pain sub-score only in this respect. A type-II statistical error may account for the discrepancy of the 6-month results for the UKR group. After 6 months there was no further statistically significant longitudinal improvement within any of the groups up to the 2-year follow-up results with the exception of the pain sub-score for the UKR group at the 1-year vs. 2-year time point. The between-group statistical analysis shown in Table 5 revealed that pre-operatively there was only a significant difference between the UKR group and the TKR group for the function sub-score. Post-operatively there was no significant difference of the mean WOMAC sub-scores between any of the three groups at any time point.

**Table 4:** Within-group statistical analysis of Western Ontario and MacMaster Universities Osteoarthritis Index (WOMAC) sub-scores

	Pre-op vs. 6 months		6 months vs. 1 year		1 year vs. 2 years	
	*p* value	95%CI	*p* value	95%CI	*p* value	95%CI
UKR^a^						
Function	0.178	− 96.9 to 169.9	0.520	− 194.2 to 225.2	0.410	− 2.8 to 5.8
Pain	0.049^*^	0.4 to 62.9	0.374	− 112.1 to 142.1	0.041^*^	0.3 to 9.7
Stiffness	0.184	− 29.0 to 79.3	0.500	− 292.7 to 342.7	0.750	− 13.3 to − 17.3
BKR^b^						
Function	< 0.001^*^	16.4 to 37.1	0.249	− 6.8 to 22.3	0.172	− 8.0 to 28.5
Pain	0.001^*^	17.5 to 45.0	0.080	− 0.6 to 8.1	0.903	− 28.8 to 31.3
Stiffness	0.009^*^	7.3 to 36.5	0.665	− 6.9 to 10.1	0.476	− 19.0 to 32.0
TKR^c^						
Function	< 0.001^*^	26.2 to 47.0	0.392	− 9.2 to 21.6	0.700	− 8.0 to 11.0
Pain	< 0.001^*^	27.9 to 49.3	0.407	− 10.0 to 22.8	0.580	− 8.4 to 13.4
Stiffness	< 0.001^*^	16.4 to 43.3	0.385	− 11.4 to 27.0	0.306	− 7.7 to 20.1

Paired Student’s *t* test

*95%CI* confidence interval of mean difference

^*^Statistically significant at  <0.05 level.

^a^*UKR* unicompartmental knee replacement group (6 months: *n* = 25; 1 year: *n* = 18; 2 years: *n* = 9)

^b^*BKR* bicompartmental knee replacement group (6 months: *n* = 41; 1 year: *n* = 29; 2 years: *n* = 16)

^c^*TKR* total knee replacement group (6 months: *n* = 45; 1 year: *n* = 26; 2 years: *n* = 14)

**Table 5:** Between-group statistical analysis of Western Ontario and MacMaster Universities Osteoarthritis Index (WOMAC) sub-scores

	UKR^a^ vs. BKR^b^		UKR^a^ vs. TKR^c^		BKR^b^ vs. TKR^c^	
	*p* value	95%CI	*p* value	95%CI	*p* value	95%CI
Pre-operative						
Function	0.914	− 11.8 to 16.6	0.031^*^	1.0 to 25.7	0.073	−0.8 to 22.7
Pain	0.863	− 13.0 to 18.5	0.435	− 6.3 to 19.8	0.793	−9.3 to 16.2
Stiffness	0.851	− 14.6 to 23.1	0.575	−9.4 to 22.9	0.921	−13.2 to 18.3
6 months						
Function	0.808	− 18.3 to 31.1	0.706	− 15.9 to 31.6	0.966	− 15.6 to 12.6
Pain	0.275	− 7.7 to 35.7	0.227	− 6.4 to 35.2	0.997	− 13.0 to 13.7
Stiffness	0.275	− 7.9 to 36.5	0.279	− 7.6 to 34.8	0.991	− 14.3 to 12.9
1 year						
Function	0.589	− 11.2 to 26.5	0.349	− 7.6 to 28.4	0.920	−14.2 to 19.7
Pain	0.498	− 9.0 to 24.8	0.328	− 6.5 to 25.7	0.961	− 13.5 to 16.9
Stiffness	0.686	− 13.8 to 28.3	0.508	− 10.8 to 29.4	0.963	− 16.9 to 21.0
2 years						
Function	0.197	− 6.7 to 40.6	0.850	− 19.4 to 30.4	0.424	− 10.8 to 33.8
Pain	0.522	− 12.8 to 33.6	0.990	− 23.0 to 25.8	0.396	− 10.2 to 33.6
Stiffness	0.304	− 11.0 to 45.6	0.929	− 25.3 to 34.2	0.472	− 13.9 to 39.5

One-way analysis of variance (ANOVA) and Tukey’s post-hoc pairwise comparison

*95%CI* confidence interval of mean difference

^*^Statistically significant at <0.05 level.

^a^*UKR* unicompartmental knee replacement group (6 months: *n* = 25; 1 year: *n* = 18; 2 years: *n* = 9)

^b^*BKR* bicompartmental knee replacement group (6 months: *n* = 41; 1 year: *n* = 29; 2 years: *n* = 16)

^c^*TKR* total knee replacement group (6 months: *n* = 45; 1 year: *n* = 26; 2 years: *n* = 14)

## Discussion

The principle finding of this study is that the clinical outcome of medial segmental BKR is comparable to that of both medial UKR and TKR. In general, there was a significant improvement of the clinical outcome scores in all three groups at 6 months following surgery as compared to the pre-operative findings. No further significant improvement was found thereafter up to the 2-year results. We found that the majority of appreciable clinical improvement occurs within the first 6 months following surgery and subsequent to that only modest improvements continue to occur. Post-operatively no significant difference was found between the three groups in terms of outcome scores. However, pre-operatively the mean scores of the TKR group were poorer than those of the UKR and BKR groups. This can be explained by the fact that the subjects who underwent TKR had more generalised (and, therefore, symptomatically more pronounced) arthritic changes in their knee as compared to the more localised and confined disease pattern considered suitable for UKR and BKR intervention.

The use of patient-reported outcome measures (PROMs) is strongly encouraged by the Department of Health [23] as a ‘means of assessing effectiveness of care’. Their use is important in both clinical practice and clinical research for measuring improvement in pain and function after surgery and for the comparison of different clinical interventions [24]. We used two validated patient-completed instruments in our study. Clinician-completed outcome scoring systems have the potential for interviewer bias and may also incur cost of resources consumed in terms of clinic and consultation time. The advantages of patient-completed knee scores include a longer time period for patients to answer the questions asked within each item of the instrument and the increased willingness to admit to unfavourable responses as interview bias is obviated. Their main disadvantage is the increased potential for missing data due to omitted responses. The primary weakness of this study was the number of missing data points post-operatively, mainly in the UKR group, due to loss to follow-up which rendered one aspect of the OKS statistical analyses too underpowered to calculate. However, the use of two validated scoring systems and the relatively high number of subjects included overall in the study (particularly in the BKR group) meant that enough data was present to adequately investigate the principle aim of the study. Loss to follow-up is common in clinical studies and can be due to many reasons including patients relocating away from the original hospital in which they had their surgery (due to retirement or moving away for work commitments) while other patients may feel that their affected knee has substantially improved and no longer have time to take off work and attend the hospital for clinic review. Other limitations of the study include different disease pattern involvement pre-operatively which then led to the use of different implants as well as non-randomisation of the subjects. However, this was a pragmatic clinical study in which patients underwent the procedure considered most appropriate for them in terms of disease severity and pattern involvement of their knee and also the prosthesis with which the operating surgeon had the most experience with. The follow-up time period was up to 2 years post-operatively. The findings of this study should, therefore, be interpreted as the short-term clinical results of a large series of medial segmental BKRs. The outcome variables of interest measured in the present study focussed on clinical outcome scoring systems and revision rates. Future studies may wish to also consider including radiological evaluations, operative time, length of hospital stay and any-cause complications.

UKR is successful in managing patients with single compartment arthritis with intact cruciate ligaments [25]. Both younger and more active patients as well the increasingly ageing population who present with symptomatic osteoarthritis of the knee have high expectations of not only improvement of pain but also physical function following surgery in order to fulfil an active lifestyle after retirement [26]. Some studies have shown that the rate of return to low-impact sporting activities (i.e. golf, bowling, cycling, swimming, etc.) was as high as 96% following UKR and as low as 63% following TKR [27]. Patients who underwent UKR were also found to return to sporting activities more quickly (mean 3.6 months) than those who had undergone TKR (mean 4.1 months). UKR and BKR retain both cruciate ligaments (and, therefore, the mechanoreceptors within them) and so preserve and maintain knee proprioception. Patient satisfaction and functional outcome have been closely correlated with knee proprioception [28]. High patient expectations and functional demands following arthroplasty may be met more readily with UKR and BKR than TKR. Approximately half of all patients who have degenerative arthritis of the knee will also have involvement of the PFJ. Symptoms include crepitus and anterior knee pain exacerbated by exercise, kneeling, squatting and stair climbing (particularly ascending stairs) [29]. UKR may be considered in a select group of patients with co-existing but strictly *asymptomatic* grade-III PFJ arthritis. Intra-operative trimming of the patella with excision of osteophytes or partial lateral facetectomy of the patella can be performed at the time of the UKR operation. However, UKR alone is not advisable in single tibiofemoral compartment arthritis with associated *symptomatic* and generalised PFJ arthritis [2, 3]. Although BKR is a more intricate undertaking than UKR, it is a less invasive surgical option than TKR in patients with associated symptomatic PFJ arthritis [30]. Furthermore, patients who have previously undergone isolated UKR or PFJ replacement and subsequently develop progressive arthritis in an unresurfaced compartment can be effectively managed by staged modular resurfacing of the newly affected compartment (provided the original prosthesis remains functional and well fixed), and thereby forestalling conversion to TKR and preserving a future arthroplasty option [31, 32].

Retaining both cruciate ligaments not only confers a proprioceptive advantage but also preserves the physiological and kinematic function of the knee joint [33, 34]. Cadaveric studies [33, 34] have shown that cruciate-sparing prostheses (i.e. UKR) demonstrate normal kinematics and compressive forces within the PFJ as compared to the native knee. However, in cruciate-sacrificing prostheses (i.e. TKR) there is loss of the physiological roll-back mechanism of the femur which induces altered kinematics and abnormally high PFJ compressive forces, especially in flexion. This adverse effect was more pronounced in posterior-cruciate-retaining TKR prostheses than posterior-stabilised ones as in the latter the polyethylene tibial peg acted as a mechanical interlock which prevented posterior subluxation of the tibia as the knee flexed. The extensor mechanism is, therefore, vulnerable to the ‘knock-on effect’ of abnormal tibiofemoral movement, with resultant changes in PFJ forces and tracking. Further in-vitro studies [35] investigating the kinematics of the tibiofemoral joint have shown that the translational and rotational knee-joint kinematics of BKR resemble that of the native knee and were indeed found to be superior to that of posterior-cruciate-retaining TKR. In-vivo studies [36, 37] evaluating knee-joint kinematics throughout a spectrum of activities of daily living motor tasks (i.e. walking, stair climbing, etc.) have shown that knee-joint kinematics in patients with unilateral BKR replicate the kinematics of their contra-lateral non-involved limb and also that of healthy external controls. Wang et al. [38] evaluated knee strength and biomechanics during walking in patients with BKR, TKR and healthy control subjects. They found that BKR patients reported higher satisfaction while performing activities of daily living and improved their quadriceps strength to a level that was close to healthy controls. However, the TKR patients had a reduced walking speed consequent to deficits in quadriceps strength and decreased extensor moment.

There have been variable reports published in the literature regarding the Journey Deuce BKR prosthesis (Smith & Nephew, Memphis, TN, USA). Its monolithic design (monobloc) consists of a combined femoral shield with a medial condylar component that resurfaces both the medial and patellofemoral (trochlear) compartments of the distal femur. This component is available as either oxidised zirconium or cobalt chrome. The medial tibial component consists of a fixed-bearing, unicondylar, metal-backed prosthesis. The patella can be left either unresurfaced or (more commonly) resurfaced with a polyethylene button [39, 40]. Some studies [40] using this prosthesis demonstrated early discharge from hospital (2 days post-operatively), good range of knee movement, easier post-operative rehabilitation, reduced intra-operative blood loss with no patients requiring blood transfusion, reduced pain and a high level of patient satisfaction. Rolston et al. [41] also showed that the Journey Deuce BKR was effective in terms of restoring the mechanical axis and correct knee alignment to the centre of the tibial plateau in 95% of cases. However, Palumbo et al. [16] reported their experience with the Journey Deuce BKR and found an overall survival rate as low as 86% at 2 years following surgery and with 31% of patients unsatisfied due to inconsistent pain relief and unacceptable functional results. Other studies [42] also found significantly higher early complications (mainly persistent pain) that required revision arthroplasty. Muller et al. [43] found that in their series of 43 cases, 18% of patients had to undergo revision within the first year due to persistent knee pain and instability. The high revision rates in these studies maybe a reflection of the technical complexity of the BKR concept. However, specifically to the Journey Deuce prosthesis itself, adverse factors may include poor implant design, insufficient variety of implant sizes and crude instrumentation. The monobloc femoral component may only allow limited positioning options, thereby making it less accommodating to the variability of the patient’s native knee (both in the medial and PFJ compartments) and, therefore, increasing the risk of component malalignment. We used a segmental modular approach (UKR with PFJ replacement) in our study that allowed separate positioning of the implants and so better individual tailoring to the patient’s specific knee anatomy. Heyse et al. [14] retrospectively reported on a series of nine BKR cases (segmental UKR with PFJ replacement) at a mean follow-up time period of 12 years with a successful outcome and high patient satisfaction and no revision procedures within the study time frame. Parratte et al. [13] reported on 77 BKR cases (segmental UKR with PFJ replacement) and found that the procedure reliably alleviated pain, improved function and restored limb alignment. The prosthesis survivorship at 17 years was 54% (27 cases were revised), of which 20 cases were for aseptic loosening of the PFJ prosthesis. Their high revision rates were attributed to the use of early generation implants, poor instrumentation and lack of experience in PFJ replacements at the time of original surgery. Future studies are, therefore, required to assess the long-term outcome of segmental BKR with modern implant designs, improved surgical techniques and appropriate patient selection.

## Conclusion

The magnitude of clinical improvement following knee replacement is greatest at 6 months; thereafter, only modest improvements continue to occur. This study found no significant differences of OKS and WOMAC at 2 years after surgery among UKR, BKR and TKR. BKR is a good alternative option for combined symptomatic medial and patellofemoral arthritis of the knee.

## Data Availability

The datasets used and/or analysed during the current study are available from the corresponding author on reasonable request.
